# The *Drosophila* homologue of *MEGF8* is essential for early development

**DOI:** 10.1038/s41598-018-27076-y

**Published:** 2018-06-08

**Authors:** Deborah L. Lloyd, Markus Toegel, Tudor A. Fulga, Andrew O. M. Wilkie

**Affiliations:** MRC Weatherall Institute of Molecular Medicine, Radcliffe Department of Medicine, University of Oxford, Oxford, OX3 9DS UK

## Abstract

Mutations of the gene *MEGF8* cause Carpenter syndrome in humans, and the mouse orthologue has been functionally associated with Nodal and Bmp4 signalling. Here, we have investigated the phenotype associated with loss-of-function of *CG7466*, a gene that encodes the *Drosophila* homologue of *MEGF8*. We generated three different frame-shift null mutations in *CG7466* using CRISPR/Cas9 gene editing. Heterozygous flies appeared normal, but homozygous animals had disorganised denticle belts and died as 2^nd^ or 3^rd^ instar larvae. Larvae were delayed in transition to 3^rd^ instars and showed arrested growth, which was associated with abnormal feeding behaviour and prolonged survival when yeast food was supplemented with sucrose. RNAi-mediated knockdown using the *Gal4*-*UAS* system resulted in lethality with ubiquitous and tissue-specific Gal4 drivers, and growth defects including abnormal bristle number and orientation in a subset of escapers. We conclude that *CG7466* is essential for larval development and that diminished function perturbs denticle and bristle formation.

## Introduction

**Figure 1:**
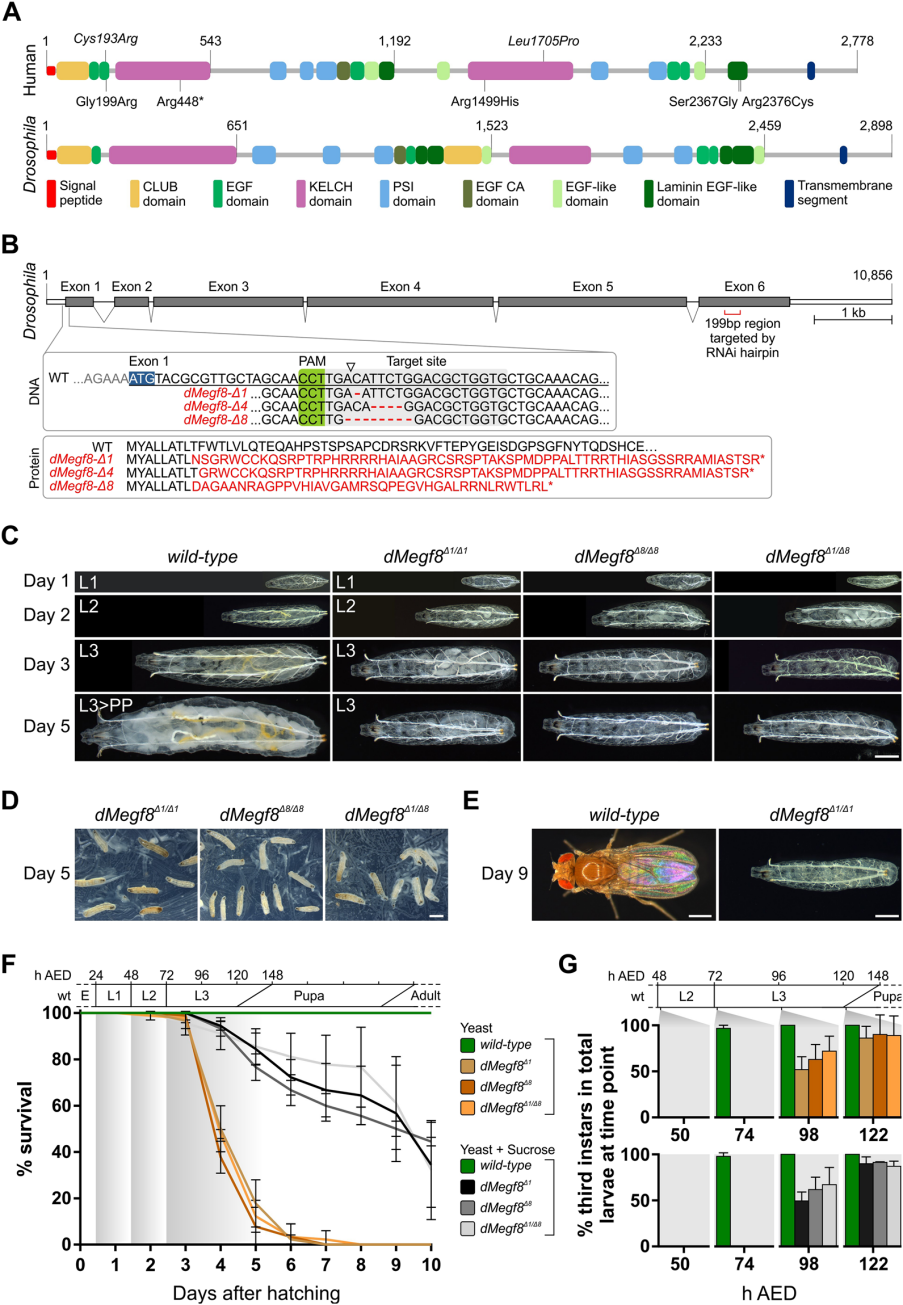
Domain organisation of the human MEGF8 and *Drosophila* dMegf8 proteins and characterisation of the *dMegf*8 null mutant phenotype. (**A**) *Top*: Domain organisation of the human MEGF8 protein (based on Uniprot reference Q7Z7M0). Shown above the cartoon are the equivalent positions of two missense mutations (italicised) identified in mouse ethylnitrosourea-induced mutants^7,8^ (note that the p.Leu1705Pro substitution is based on the numbering for the mouse Uniprot reference P60882 but appears as p.Leu1775Pro in the original report)^7^. Below the cartoon are amino acid substitutions identified in *MEGF8*-Carpenter syndrome patients^4^ (upright text), including a previously unreported substitution (c.7126 C > T encoding p.Arg2376Cys, identified in *trans* with c.7068 + 5 G > A; C.J. Curry, A.O.M.W., unpublished). *Bottom*: Domain organisation of the *Drosophila* MEGF8 ortholog CG7466 (dMegf8). Note the high degree of domain conservation. (**B**) The *dMegf8* gene showing the CRISPR-Cas9 genomic target site and the mutations generated by this approach (top box, triangle indicates Cas9 cleavage site) and the predicted effect of the mutations on the encoded protein, including the early termination by the three frameshift deletions (bottom box), along with the location in exon 6 of the 199 bp hairpin that targets *dMegf8* mRNA for degradation via RNAi. (**C**) Differences in morphology become apparent at larval day 3. The *dMegf8* null mutants exhibit a growth arrest after larval day 3, with no size difference apparent between days 3 and 5. This contrasts with the significant growth in the wild-type larvae during the same time period. Note that the Malpighian tubules in the null mutants lack the characteristic yellow colour due to the genetic background (*w*−). L1 – first larval instar, L2 – second larval instar, L3 – third larval instar, L3 > PP – prepupa. (**D**) Images of agar plates with *dMegf8* mutant larvae arrested in development. Most larvae have died by day 5. (**E**) *Wild-type* animals have reached the adult stage by day 9 while the few surviving *dMegf8* mutants are still larvae. (**F**) Viability curves for d*Megf8* mutants reared on agar plates supplemented either with wet yeast paste or wet yeast paste and sucrose. Mutants arrested at larval stages 2 or 3 while wild-types progressed through the developmental stages depicted on top of the graph. E – embryonic stage, L1 – first larval instar, L2 – second larval instar, L3 – third larval instar. *n* = 90; error bars indicate standard deviation between the three replicate plates (30 animals per plate) for each fly line. (**G**) The transition from 2^nd^ to 3^rd^ instar was delayed in *dMegf8* null mutants. At the start of day 3 (74 h AED), >97% of wild-type larvae were 3^rd^ instars but all null mutants were still 2^nd^ instars. In *dMegf8*^*Δ/Δ*^ mutants, the transition to 3^rd^ instar occurred during days 4 or 5 in most larvae. Scale bars: a = 0.5 mm, b = 1 mm, c = 0.5 mm. *n* = 90, error bars indicate standard deviation between the three replicate plates (30 animals per plate) for each fly line.

Over the past 25 years, investigation into the genetic basis of multiple congenital abnormality syndromes has provided a powerful route to the discovery and functional analysis of novel genes with pleiotropic roles in embryonic development. One such disorder, Carpenter syndrome (first described in 1901)^1^, is characterised by a combination of craniosynostosis (premature fusion of the cranial sutures) and polysyndactyly of the hands and feet. Other frequent features of this disorder include hypogenitalism, congenital cardiac defects, umbilical hernia and learning disability^2^. Carpenter syndrome is most frequently caused by biallelic mutations in *RAB23*^3^, which encodes a small guanine nucleotide binding protein involved in vesicle transport. More recently, it was reported that patients with Carpenter syndrome who are negative for *RAB23* mutations harbour biallelic mutations in the Multiple Epidermal Growth Factor-like Domains 8 (*MEGF8*) gene^4^. Patients with *MEGF8* mutations share many of the features of *RAB23*-mutated individuals, but disorders of left-right laterality are more frequent. *MEGF8* encodes a multi-domain protein (Fig. 1A) conserved in many metazoan species, with similarities to Attractin, which functions in trafficking membrane-bound receptor molecules either to the cell surface or to the lysosome for degradation^5^.

Providing some clues to the biological role of MEGF8, recessive mutations encoding missense substitutions (Fig. 1A, annotated above cartoon of human protein) in the murine orthologue *Megf8* result in developmental defects similar to the human disorder, including skeletal deformities and abnormal left-right (L-R) patterning^6,7^, and have led to proposed roles for *Megf8* in Nodal^8^ and BMP^7^ signalling. Interestingly, recessive, loss-of-function (LOF) mutations in murine *Rab23* also cause left-right patterning defects^9^. As laterality defects frequently arise as a consequence of ciliopathies^10^, it is noteworthy that both *Rab23* and *Megf8* mutant mouse embryos with left-right patterning defects have fully motile cilia that generate the leftward nodal flow^8,9^ required during early symmetry breaking^11^. In addition to patterning defects, *Megf8* knockout and LOF mouse embryos exhibit disrupted axon guidance in the peripheral nervous system, indicating a role for *Megf8* as a mediator of BMP4^7^. It is notable that BMP antagonism is required to facilitate the establishment of the L-R axis^12^, defects of which are a hallmark of *Megf8* mutations. Thus, the action of MEGF8 on signalling by multiple members of the TGF-β/BMP family could explain the phenotypic spectrum that results from mutations in this gene. Collectively this work suggests that both Rab23 and Megf8 may be involved in trafficking cargoes in similar cellular processes.

A strong homology between vertebrate MEGF8 and the protein encoded by the *Drosophila melanogaster* gene *CG7466*, hereafter referred to as *dMegf8*, has been noted previously^4^. For example, the linear homology between dMegf8 and human MEGF8 can be traced over >2,400 amino acids, including 33% identities, and the proteins display extensive domain conservation (Fig. 1A). This conservation, combined with the tractable nature of *Drosophila* genetics, provides a potential model system in which to study the cellular biology and function of MEGF8.

Little prior work has been performed on *dMegf8*, although it has been highlighted as potentially significant in genetic screens investigating various developmental and behavioural pathways. In two genome-wide RNAi-screens (one for regulators of the Notch pathway^13^ and one for control of ecdysone signalling)^14^, *dMegf8* knockdown resulted in a cell death/reduced cell viability phenotype, although there was no apparent connection between *dMegf8* and the pathways under investigation. Two further genome-wide RNAi screens (one for novel genes involved in heat nociception^15^ and one for pathways involved in the cardiovascular system)^16^ did not report a phenotype with *dMegf8* knockdown. In a small-scale overexpression screen *dMegf8* was reported to have the strongest effect in suppressing the mesodermal migration defects in Pebble (*pbl*) mutants^17^, indicating a potential role for dMegf8 in mesoderm development. A yeast-2-hybrid screen to identify interaction partners for Lawc, a protein required for proper transcription by RNA polymerase II, detected *dMegf8*, although this was at a low frequency^18^. Three studies examining the genetic basis of feeding and olfactory behaviour identified *dMegf8* as a putative candidate^19–21^. Additionally *dMegf8* was amongst ~900 *Drosophila* genes predicted by a machine-learning approach to contribute to synaptic assembly and function^22^, potentially reminiscent of the role of murine Megf8 in axon guidance^7^.

The identification of *dMegf8* in these screens suggests important biological roles, but its LOF phenotype has not previously been investigated. Here we find that *dMegf8* is essential for *Drosophila* viability and LOF results in lethality during larval stages. The *dMegf8* larval mutant phenotype is similar to that of some *Drosophila* BMP-signalling mutants and exhibits disturbances in denticle and bristle formation. The understudied nature of this gene is reflected in the paucity of *Drosophila* reagents such as mutant lines and deficiencies, which currently hinder the use of the fly as a model to explore the function of Megf8. To address this limitation, we have generated reagents, including null mutants and a cDNA clone, which will be of use in further investigations.

## Results

### Homozygous *dMegf8* null mutants are embryonic viable but die as larvae

To explore the *in vivo* consequences of *dMegf8* LOF, we used CRISPR-Cas9 gene editing to generate three independent null mutant stocks (*dMegf8*^*Δ*1^, *dMegf8*^*Δ4*^, *dMegf8*^*Δ8*^) (Fig. 1B). Whilst heterozygous flies were fully viable and phenotypically indistinguishable from the wild-type, we found that homozygous mutations in *dMegf8* were lethal during development. *dMegf8*^*Δ1/Δ1*^ and *dMegf8*^*Δ8/Δ8*^ homozygous null mutants, as well as *dMegf8*^*Δ1/Δ8*^ *trans*-heterozygotes, (collectively referred to in further experiments as *dMegf8*^*Δ/Δ*^) were embryonic viable, hatching into first instar larvae and undergoing the first moult into second instars within the same timeframe as wild-type flies (Fig. 1C), but became growth arrested (Fig. 1C–E) and began to die from day three (74 h after egg deposition - AED) onwards (Fig. 1F). The majority of *dMegf8*^*Δ/Δ*^ mutants died in a short period of time (48 h) between larval days three and five (Fig. 1F), although ~2% survived for up to 20 days when their yeast diet was supplemented with sucrose (see below).

### *dMegf8* null mutants exhibit a delayed transition to 3^rd^ instar and arrest growth after day 3

At equivalent developmental times during the late 2^nd^/early 3^rd^ instar stages onwards, *dMegf8*^*Δ/Δ*^ larvae appeared morphologically distinct from their wild-type counterparts. Firstly, the mutant larvae were developmentally delayed in their transition to 3^rd^ instar (Fig. 1G). At the start of larval day three (quantified at ~74 h AED), >97% of wild-type larvae were already 3^rd^ instars whereas no *dMegf8*^*Δ/Δ*^ larvae of the same age had made this transition. Of the *dMegf8*^*Δ/Δ*^ larvae still living, 3^rd^ instars accounted for only ~50–70% by the start of larval day four (~98 h AED) and ~80–90% by the start of larval day five (~122 h AED). Both 2^nd^ and 3^rd^ instars were found among the dead larvae. Secondly, *dMegf8*^*Δ/Δ*^ larvae exhibited a growth arrest after larval day three (Fig. 1C; compare wild-type to *dMegf8*^*Δ/Δ*^ sizes on larval day five and the mutant sizes on larval days three and five).

### An abnormal feeding behaviour modified by feeding preferences is present in *dMegf8* mutant larvae

**Figure 2:**
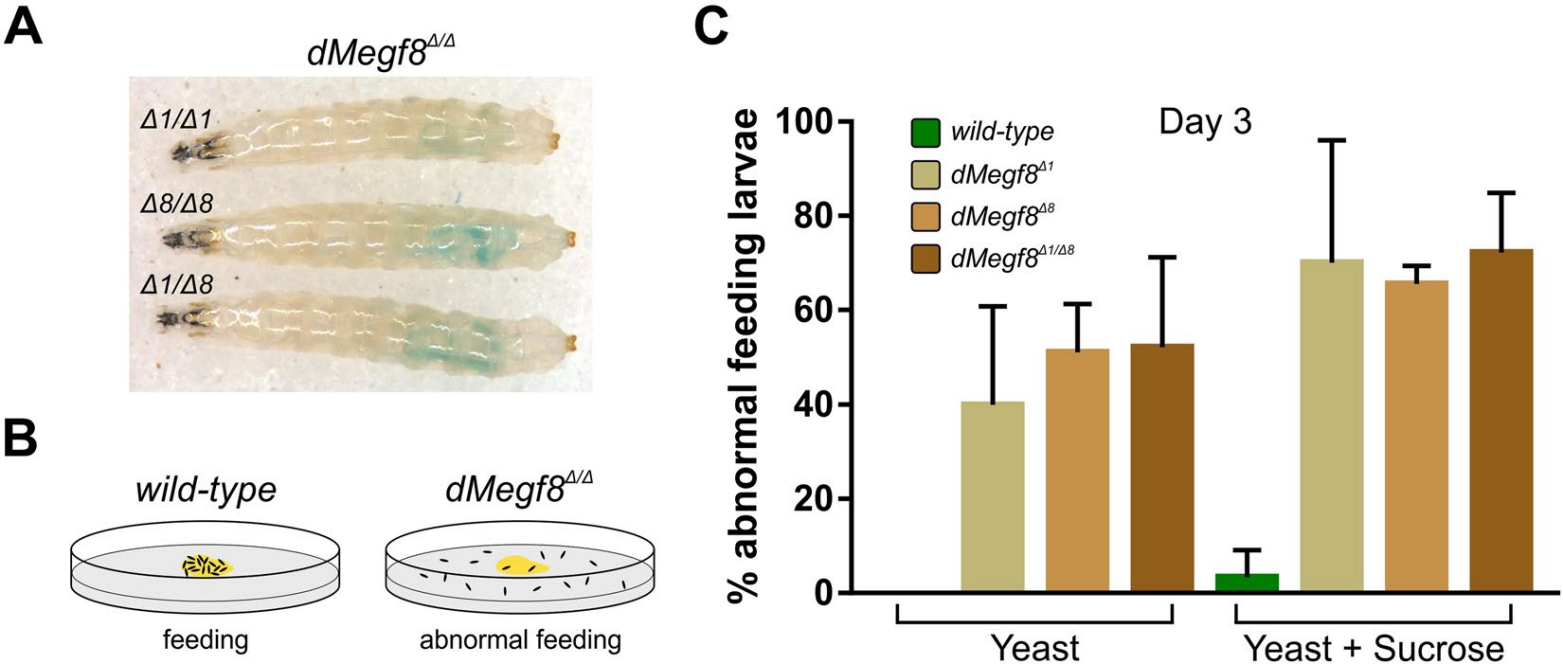
Feeding behaviour of dMegf8 mutant larvae. (**A**) Food coloured with blue-dye is evident in the intestinal tract of 3-day old *dMegf8*^*Δ/Δ*^ larvae. Note that although it is larval day 3 these mutants are still 2^nd^ instars. (**B**) *dMegf8* mutants exhibited abnormal feeding behaviour, as illustrated in this snapshot cartoon. The black dots represent larvae and the food source is depicted in yellow. (**C**) Quantification of the abnormal feeding phenotype of larvae fed on yeast (left) and yeast + sucrose (right). Thirty 1^st^ instar larvae were placed on agar plates containing either yeast or yeast + sucrose and the number of larvae outside the food source counted 48 hours later; error bars show standard deviation between 3 replicate plates.

As slowed development and growth arrest may be secondary to starvation, we excluded the possibility that these phenotypes were caused by the inability of *dMegf8* mutant larvae to ingest food using a feeding assay; food was clearly visible in the larval gut (Fig. 2A). For the majority of the larval phase (five to six days), *Drosophila* larvae exhibit a foraging behaviour in which they remain buried in the food source, eating continuously until they reach a critical mass for pupation, whereupon they display a wandering behaviour in which they stop feeding and exit the food to search for a suitable pupation site^23^. Unlike wild-type larvae of the same age, *dMegf8*^*Δ/Δ*^ mutants displayed an abnormal feeding behaviour, leaving the food (wet yeast paste, normally a strong attractant) as early as larval day two. To quantitate this phenotype, we performed an assay on day three which revealed that, out of 30 individuals, an average of ~40% *dMegf8*^*Δ1*^, ~51% *dMegf8*^*Δ8*^ and ~52% *dMegf8*^*Δ1/Δ8*^ larvae were outside the food source in comparison to 0% of the wild-type larvae (Fig. 2B,C, left panel).

Given that the null mutants initially move with the same vigour as the wild-type larvae and bury themselves in the food for the first day or two, we considered reasons other than general illness or an inability to perceive food to explain this abnormal feeding behaviour.

### The viability of *dMegf8* mutants is increased by feeding yeast supplemented with sucrose

The starvation-like phenotype of the *dMegf8* mutants is similar to that of larvae reared under hypoxic conditions^24^, raising the possibility that hypoxia could be the basis of the d*Megf8* mutant phenotype. Supplementing the yeast food source with sucrose was shown to modify the food-avoidance behaviour of hypoxic flies and consequently increase their lifespan^25^. To test whether the addition of sucrose encouraged *dMegf8* mutant larvae to remain foraging, we repeated the feeding assay on agar plates with either wet yeast paste (Y) or wet yeast paste supplemented with sucrose (Y + S). However, the number of larvae outside the food source was increased on the Y + S plates (Fig. 2C), including a slight increase in the frequency of wild-type larvae found outside the yeast paste (wild-type ~3.33%; ~70% *dMegf8*^*Δ1*^; ~65% *dMegf8*^*Δ8*^; ~72% *dMegf8*^*Δ1/Δ8*^). A possible explanation is that leaching of the sucrose-solution out of the Y + S paste formed wetter conditions around the periphery, which potentially served as a source of sucrose that was separate from the yeast. Supporting this explanation, we noted that on the Y + S plates larvae outside the food source were more frequently found in this halo compared with a more random distribution on the Y-only plates. Mutant larvae fed on Y + S were also observed moving in and out of the food, suggesting that they were continuing to forage. This is consistent with results from food-choice experiments in hypoxic flies that found a preference for sucrose over yeast^25^.

To test whether adding sucrose to wet yeast paste increased the life span of *dMegf8*^*Δ/Δ*^ mutants, we performed a viability assay. Complete lethality of *dMegf8*^*Δ/Δ*^ larvae fed solely on yeast occurred between days three and six. In contrast, when fed on Y + S, >60% of *dMegf8*^*Δ/Δ*^ larvae were still alive on day seven and >30% were alive on day ten (Fig. 1F). Although mutant larvae fed on the Y + S diet remained highly active compared to those fed on yeast alone, the addition of sucrose did not rescue the growth arrest; larvae fed on Y + S did not pupate and remained arrested in the 3^rd^ instar larval stage for an extended period (some for >20 days) before death.

### *dMegf8*^*Δ/Δ*^ mutants have denticle belt phenotypes suggestive of a defect in polarity

**Figure 3:**
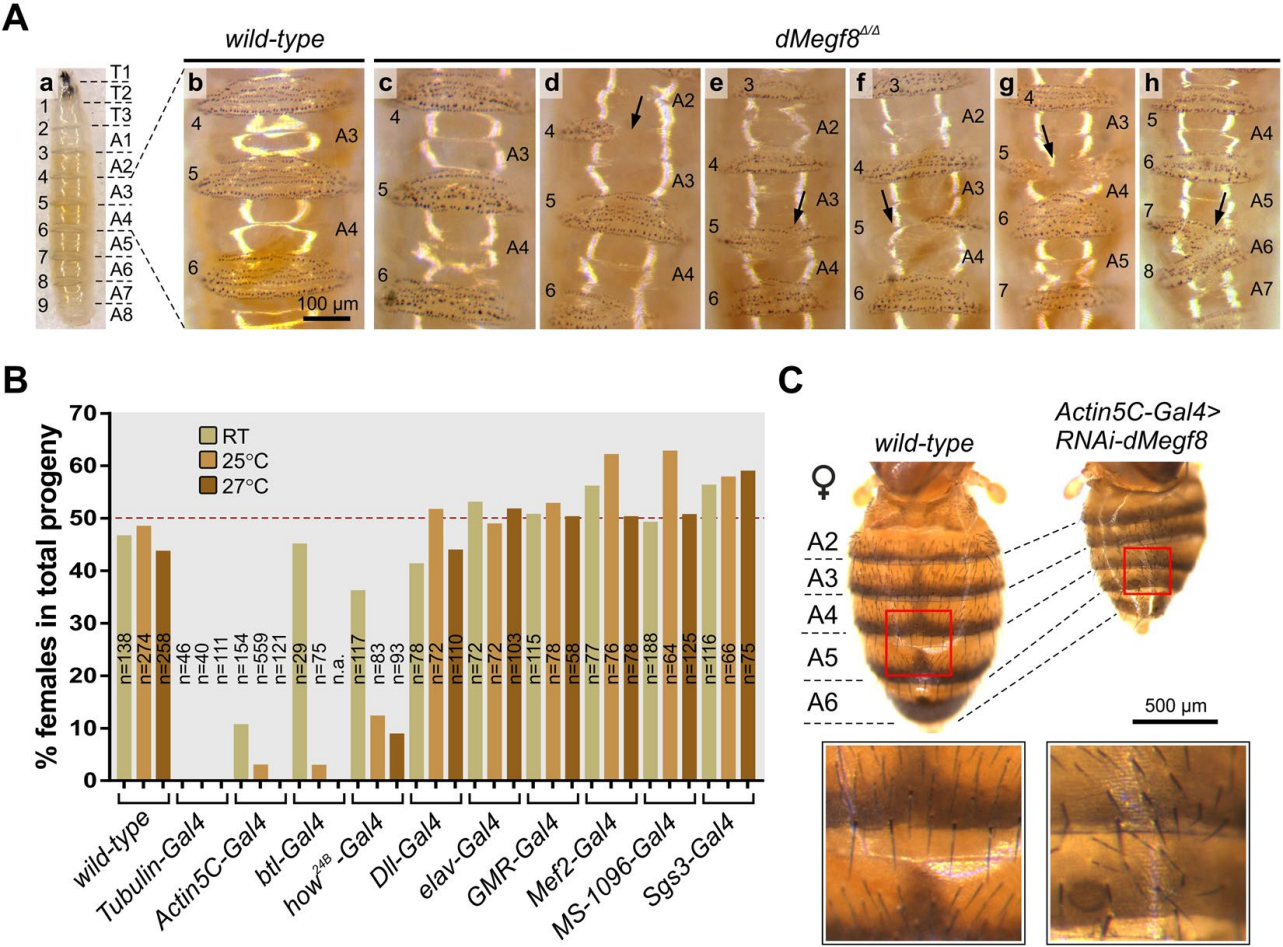
Denticle belt defects in dMegf8 null mutants and characterisation of the RNAi knockdown phenotype. (**A**) The larval ventral cuticle is covered in nine belts of denticles (a). In the wild-type each belt has seven rows of denticles (b), the number and orientation of which are controlled by Wnt and PCP signalling. Denticle belt defects were found on *dMegf8* mutants (c–h) with the phenotype ranging from mild (a generally disorganised belt appearance, (c), to severe (belts completely or partially missing, d–g, or fused with adjoining belts, h). Numbers at the side of each image refer to the belt/segment number. The wild-type larvae (a) was two days old, *dMegf8*^*Δ/Δ*^ larvae (b–h) were five days old. (**B**) *dMegf8* knockdown is lethal, as shown by the reduction in the number of affected adult females in progeny from crosses between *UAS-RNAi-dMegf8* males carrying the inducible UAS construct on the X chromosome and ubiquitous or restricted-expression *Gal4*-driver females. Estimated lethality of females when reared at 25 °C was highest for ubiquitous knockdown by *Tubulin-Gal4* (100%) and *Actin5C-Gal4* (~97%), followed by the more specific drivers *btl-Gal4* (~97%) and *how*^24B^*-Gal4* (~88%). Female progeny from the control crosses using wild-type instead of Gal4-driver females were close to the expected ~50%. The degree of female lethality from *dMegf8* knockdown varies with temperature due to the dose-dependent nature of the Gal4-UAS system. For all drivers, female lethality was higher at 27 °C and lower at RT. Crosses with *Tubulin-Gal4*, the strongest driver, were 100% lethal at all temperatures. Two independent crosses were performed for all drivers except *Actin5C-Gal4* and *btl-Gal4* for which 6 and 1 crosses were performed, respectively; there was no significant difference in female lethality between independent crosses for the same Gal4 driver (one-way ANOVA *p* > 0.5). *n* = the total number of progeny counted (male and female flies). (**C**) Example of bristle defects in female escapers from the mostly lethal *dMegf8* knockdown with the *Actin5C-Gal4* driver reared at 25 °C. On the dorsal abdomen of wild type flies bristles uniformly pointed posteriorly, but exhibited a disorganised appearance in ~50% of the female RNAi escapers.

In *Drosophila*, mutations in *Rab23* result in abnormal orientation and number of adult cuticular hairs, identifying a unique class of planar cell polarity (PCP) genes dedicated to regulating the planar polarization of these structures^26^. Given the phenotypic overlap arising from mutations in human *RAB23* and *MEGF8*, we examined the *dMegf8*^*Δ/Δ*^ larvae for evidence of perturbations in polarity. The ventral surface of *Drosophila* larvae normally has nine belts of denticles, which are small actin protrusions that function to provide traction for motility (Fig. 3A, panels a & b). Each belt comprises seven rows of individual denticles that point either forwards (rows 0, 1 and 4) or backwards (rows 2, 3, 5 and 6) in a process controlled by PCP^27,28^ and Wnt/wingless (wg) signalling^29,30^. Mutant larvae exhibited defects ranging from a frequently-occurring mild phenotype in which there was a generally disorganised appearance of the belts (Fig. 3A, panel c), to more severe phenotypes present in ~6% of larvae in which entire belts were partially or completely missing or fused with adjacent belts (Fig. 3A, panels d–h). These anomalies affected different belts (indicated by arrows in Fig. 3A, panels d–h), suggesting they originated from perturbations in a global pathway rather than a localised or segment-specific defect.

### RNAi knockdown of *dMegf8* results in variable lethality and escapers exhibit bristle defects

The *dMegf8*^*Δ/Δ*^ mutants are complete LOF mutants, but the human disease caused by missense mutations in MEGF8 may arise due to a window of residual function in the mutant protein^4^. As such, we explored the phenotypic consequences of reducing the amount of *dMegf8 in vivo* by using the *Gal4-UAS* system^31^ to knock down *dMegf8* in a variety of tissues and developmental stages. In the *UAS-RNAi-dMegf8* transgenic fly stock, the inducible UAS responder construct that targets a 199 bp region from exon 6 of the *dMegf8* transcript for degradation (Fig. 1B) is inserted on the X (1^st^) chromosome in male flies only. Consequently, crosses to virgin females from Gal4 driver lines result in progeny in which only the females are affected.

To determine the effect of a global *in vivo dMegf8* knockdown we used the strong ubiquitous *Tubulin-Gal4* (*Tub-Gal4*) driver. When reared at 25 °C, ~50% of the progeny from the control cross were female, but *dMegf8* knockdown with *Tub-Gal4* resulted in no female progeny; similarly, only ~2.7% female progeny were observed with another ubiquitous but slightly weaker driver, *Actin5C-Gal4* (Fig. 3B).

To investigate whether *dMegf8* knockdown in specific tissues or developmental stages resulted in lethality and/or gross morphological defects, we used publicly available expression profile data (FlyAtlas and modENCODE, accessed via FlyBase) to select drivers expressing in more restricted tissues/developmental stages likely to overlap with *dMegf8* expression. Marked lethality was observed with *breathless-Gal4*^32^ (*btl-Gal4)*, which drives expression in the tracheal- and CNS midline cells, and *how*^24B^*-Gal4*^31,33^, which drives expression in early mesoderm and mesodermally-derived tissues, muscles and CNS midline cells as well as a subset of peripheral ectodermal tissues, including larval tracheal cells, and in the dorsal neurohemal organs. *dMegf8* knockdown with these drivers resulted in females accounting for ~3% and ~12% of the total progeny, respectively (Fig. 3B). No significant lethality resulted from *dMegf8* knockdown with the following Gal4 drivers: *MS1096-Gal4*^34^ (drives expression in the wing imaginal disc), *elav*^*c155*^*-Gal4*^35^ (CNS driver), *Mef2-Gal4*^36^ (muscle driver), *Dll-Gal4* (distal appendages driver), *GMR-Gal4* (commonly used as an eye driver but also expressed in other larval tissues)^37^, and *Sgs3-Gal4* (salivary gland driver, not anticipated to cause an effect based on the expression profile of *dMegf8* detecting no expression in salivary glands).

As the Gal4-UAS system is dose-dependent and Gal4 activity is influenced by temperature, it is possible to impose some control over the degree of knockdown. On rearing the crosses at two additional temperatures (27 °C and room temperature [RT], ~23 °C), we found that, for most drivers, female viability decreased at higher temperature and increased at lower temperature, supporting a dose-dependent knockdown of *dMegf8* as the cause of the reduction in female progeny (Fig. 3B). However, the strongest ubiquitous driver, *Tub-Gal4*, was 100% lethal at all temperatures.

To investigate whether *dMegf8* knockdown resulted in gross morphological phenotypes in addition to lethality, we examined the female escapers reared at 25 °C. Approximately 50% of escapers from knockdown by *Actin5C-Gal4* had defects in the number and orientation of their abdominal sensory bristles (Fig. 3C). As the number and orientation of these multicellular projections of the peripheral nervous system (PNS) are largely controlled by the two genetic systems that form PCP^38–42^, this phenotype is potentially analogous to the denticle belt defects found in the null mutants and could support a role for dMegf8 in PCP. However, these animals were escapers of lethality and exhibited symptoms of sickness, such as small size and narrow abdominal segments, which can also be associated with bristle defects, raising the alternative possibility these arose by a generalized, non-specific mechanism. No bristle defects or other gross morphological phenotypes were detected in knockdown escapers with other drivers when reared at 25 °C, or in any crosses reared at RT.

## Discussion

Despite having deep evolutionary origins and an important role in development, little is known about the function of MEGF8 other than proposed roles in Nodal^8^ and BMP^7^ signalling. In *D. melanogaster*, we studied the *in vivo* consequences of *dMegf8* null mutation and knockdown, and found that non-functional *dMegf8* results in lethality during the larval phase, revealing that this gene has an essential role in *Drosophila* development and viability.

*dMegf8* LOF mutants, generated by CRISPR-Cas9 based gene editing as three independent frameshifting mutations in exon 1 (giving rise to predicted truncated proteins of 48 to 73 amino acids compared to the full-length 2898 amino acids of the wild-type protein), resulted in a lethal phenotype. Whereas heterozygous flies appeared normal, homozygotes for the two different mutations analysed in detail (*dMegf8*^*Δ1*^ and *dMegf8*^*Δ8*^) were lethal at the larval stage, with essentially identical phenotypes. The observation of similar phenotypes in both frameshift phases excludes a substantial contribution made by the illegitimate amino acids beyond the frameshift, and the observation of the same phenotypes in compound heterozygotes (*dMegf8*^*Δ1/Δ8*^) rules out off-target effects of the gene knockout strategy. Furthermore, we also observed lethality associated with RNAi-mediated *dMegf8* knockdown using ubiquitously expressing Gal4 drivers.

In addition to generating novel tools for future studies, our characterisation of the mutant phenotype enables some speculations about possible functions of MEGF8 that will provide avenues for future investigation; first, the presence of defects suggestive of abnormal polarity and second, similarity to the phenotype associated with mutation of *gbb* (*glass-bottomed boat*), a *Drosophila* homologue of the mammalian BMP5-8 protein family^43^.

Initial evidence for a polarity defect was provided by the observation that complete loss of dMegf8 was robustly associated with defects of the larval ventral cuticle (Fig. 3A), as also seen in *dachsous* (*ds*), *frizzled* (*fz*)^28^ and *wingless (wg)*^29^ mutants. Supporting this interpretation, we also observed orientation defects of the abdominal bristles of female survivors from the *Actin5C-Gal4* knockdown (Fig. 3C). Although we cannot eliminate a general, non-specific effect as the cause of the bristle defects in the *Actin5C-Gal4 knockdown* escapers, similar defects have been observed in mutations of known PCP genes, including *starry night* (*stan*) and *ds*^41,42^, and PCP-like defects are also seen in *Rab23* mutants^26^.

Larval development is regulated by genetic mechanisms that coordinate developmental progression and systemic growth with nutrient uptake and utilisation^44^. The phenotypes exhibited by *dMegf8* mutant larvae (delayed developmental progression, growth arrest and death prior to pupation) are similar to those observed in *gbb*^43^ and *dTOR*^45^ mutants, although different in details of relative severity and progression. The *gbb*^43^ and *dTOR*^45^ phenotypes have been attributed to failure to maintain energy homeostasis during development, with their phenotypic overlap caused by signalling crosstalk.

Although the similarity of *dMegf8* and *gbb* mutants provides an appealing link to the previously suggested disturbance of BMP signalling in mice^7^, given the nonspecific nature of the early lethal phenotype, several other possibilities can be envisaged. The preference of *dMegf8* mutant larvae for a yeast plus sucrose rather than yeast-only diet, which was also associated with increased longevity, has previously been described in hypoxic flies^24,25^. A potential mechanistic connection is that dMegf8 is required for tracheal development/function. We found that RNAi knockdown of *dMeg8* using GAL4 drivers (*btl-Gal4* and *how*^24B^*-Gal4*) expressed in tracheal cells resulted in significant lethality, and transcriptomic data from modENCODE and FlyAtlas indicate high expression levels for *dMegf8* in the trachea suggesting a potential role for *dMegf8* in this organ. Other possibilities include a behavioural reduction in food intake, as previously described for knockdown of *dMegf8* with a weak, ubiquitously expressed Gal4 driver^20^, or perturbations in neuronal connectivity, such as with hyperactivation of PPK1 neurons^46^ or loss of function in gustatory neurons^47^, both of which are associated with abnormal feeding behaviour and are noteworthy given the predicted role of dMegf8 in synaptic assembly and function^22^.

In summary, this work describes the phenotypes associated with loss of *dMegf8* and provides a platform for further studies of the function of this gene using genetic and cell biology approaches. To aid such further studies, we have constructed the null mutant lines described here, along with molecular reagents that include a cDNA clone of the ~9 kb *dMegf8* gene.

## Methods

All DNA oligonucleotides used are listed in Supplementary Information 1.

### Fly stocks and maintenance

Unless otherwise specified, all stocks and crosses were maintained at 25 °C. The wild-type stock was Oregon-R.

### CRISPR-Cas9 gene editing

#### Testing the efficiency of guide RNAs

Guide RNAs were designed to target the 5′ end of the endogenous *dMegf8* gene (*CG7466* reference sequence accessed from FlyBase). Potential off-target sites within the *Drosophila* genome were identified by BLAST and the CRISPR design tool http://crispr.mit.edu. Pairs of DNA oligonucleotides containing the 20-nucleotide guide sequence plus ends complementary to BspQI-digested overhangs were annealed (10 µl each of 100 µM forward and reverse oligos + 20 µl of ddH_2_O; thermocycler program: 37 °C for 30 min, 95 °C for 5 min, ramp down 0.1 °C/s, 25 °C for 10 s). 1 µl of this oligoduplex was phosphorylated (using 1 µl T4 DNA ligase buffer, 1 µl T4 PNK, 7 µl ddH_2_O; incubated at 37 °C for 30 min) and ligated into a BspQI-linearised pAC-sgRNA-Cas9-Puro vector (Addgene #49330) containing the gRNA scaffold sequence under a dU6 promoter and the Cas9 coding sequence under an Actin5C promoter.

Plasmids containing each guide were verified by dideoxy-sequencing (BigDye, Life Technologies) then transfected into *Drosophila* S2R + cells (*Drosophila* Genomics Resource Center) as described in Bassett *et al*.^48^. In brief, S2R + cells were grown in Schneider’s medium supplemented with 10% heat-inactivated fetal bovine serum at 25 °C. For transfection, cells were plated at 2 × 10^6^ cells per well of a 6-well dish, and a total of 2 µg DNA was transfected into each well using Fugene HD (Promega) at a 1:3 ratio (µg:µl), following the manufacturer’s instructions. After three days, selection was performed in 5 µg/ml puromycin. Genomic DNA was extracted using QuickExtract solution (EpiBio) following the manufacturer’s instructions, and 1 µl was used in subsequent PCR reactions.

The presence of indels was analysed by high resolution melt analysis (HRMA) as described by Bassett *et al*.^49^. Briefly, forward and reverse primers were designed to give 100–200 nucleotide products spanning the intended Cas9 cleavage site. PCR reactions were performed with 1 µl gDNA, 5 µl Hotshot Diamond PCR mastermix (Clent Lifescience), 200 nM of each oligonucleotide and 1 µl LC Green Plus dye (Idaho Technology). Cycling conditions consisted of a 5-minute denaturation step at 95 °C followed by 45 cycles of {95 °C for 20 s, primer T_m_ for 30 s, 72 °C for 30 s}, 95 °C for 30 s, 25 °C for 30 s, 10 °C hold. Thermal melt profiles were collected on a LightScanner (Idaho Technology) (70–98 °C, hold 67 °C) and analysed with the LightScanner Call-IT software. PCR products were purified and cloned into pGEM®-T Easy (Promega). Five colonies for each PCR product were grown overnight in LB with Ampicillin selection, and plasmid DNA extracted via miniprep followed by dideoxy-sequencing (using the respective HRMA oligos) to confirm the presence and type of indel.

#### Fly null mutant generation via CRISPR-Cas9

For the generation of *dMegf8* fly mutants via CRISPR-Cas9, plasmids were prepared for microinjection into fly embryos. Oligonucleotides containing the selected guide sequence were redesigned with homology to BbsI-overhangs, annealed and phosphorylated as described above and cloned into the pCFD3-dU6:3 vector (Addgene #49410). Positive colonies were identified by colony PCR and the guide sequence insertion verified by dideoxy-sequencing. Verified plasmids were extracted via maxiprep from an overnight culture and 20 µl of a 1 µg/µl preparation sent to the Cambridge Fly Facility for microinjection into fly embryos.

As we anticipated null mutations may be lethal, plasmids were injected into *nos-Cas9* embryos (Bloomington #54591: *y*^*1*^ *P*(*nos-cas9, w*+) M(3xP3-RFP.attP)ZH-2A w*) in which Cas9 expression is restricted to the germline^50^. Surviving larvae were returned to us by the Cambridge Fly Facility. Males and virgins were collected as they eclosed and used in the crossing strategy given in Bassett *et al*.^51^. In short, eclosed adults were crossed to *Sco*/*CyO* (BL #2555) balancer line virgins or males. After ~5 days, potential mosaic mutant parents were removed from successful crosses and genomic DNA extracted from the whole fly via a standard squish protocol (see below). 1 µl of DNA was used in PCR reactions to amplify the region around the CRISPR-Cas9 target site prior to dideoxy-sequencing to identify indels. Individual progeny from crosses involving a mutation-positive parent were crossed to *Sco/CyO* and after ~5 days DNA was extracted from a single wing squish (see below) followed by PCR and dideoxy-sequencing to confirm inheritance of the parental mutation. Male and virgin progeny from mutation-positive flies were then crossed to each other to generate a mutant stock balanced over *CyO*.

To identify homozygous null mutants during the viable embryo and larval stages a “red” balancer carrying mCherry (ChFP) under the control of the *squamous* promoter was used. To generate the balanced mutant lines, white-eyed heterozygote *dMegf8*^*Δ1*^*/CyO* and *dMegf8*^*Δ8*^*/CyO* virgins were crossed to red-eyed *Sco/CyO, ChFP* (BL#35523) males. Red-eyed virgin and male progeny were collected and crossed to each other to generate stable *dMegf8*^*Δ1*^*/CyO, ChFP* or *dMegf8*^*Δ8*^*/CyO, ChFP* stocks from which homozygous null mutant embryos and larvae could be identified. Transheterozygous null mutants were generated by crossing *dMegf8*^*Δ1*^*/CyO, ChFP to dMegf8*^*Δ8*^*/CyO, ChFP* and selecting non-Cherry progeny.

*Sco*/*CyO* and *Sco*/*CyO, ChFP* (BL #35523: *w*^1118^*; sna*^*Sc*^*°/CyO, P{sChFP}2*) were used to maintain the homozygous lethal *dMegf8* null mutant stocks. General information on balancer chromosomes can be found at http://flystocks.bio.indiana.edu/Browse/balancers/balancer_intro.htm. Further details on “red balancers” such as *Sco*/*CyO, ChFP* can be found at http://flybase.org/reports/FBrf0213431.html.

### Genomic DNA extraction (squish protocol)

Genomic DNA was extracted from single flies or single wings by homogenising in 50 μl or 10 μl squishing buffer (10 mM Tris-HCl, pH 8.2, 1 mM EDTA, 25 mM NaCl, 200 μg/ml proteinase K (NEB #P8102)), and heating to 37 °C for 30 minutes, followed by inactivation at 95 °C for 2 min (see Carvalho *et al*.^52^).

### Fly embryo collection

Adult flies were anaesthetised with CO_2_, transferred to embryo collection cages, and given 1–3 days to acclimatise prior to collections. Embryos were collected on fruit agar plates (100 ml grape juice, 100 ml water, 2 g agar) with a source of wet yeast paste. Two 30-minute pre-lays were performed prior to collection and collections were limited to two hours to ensure all larvae were of similar age. Embryos and larvae were aged at 25 °C.

### Null mutant viability and larval transition assays

Twenty-four hours after the embryo collection, 1^st^ instar larvae were picked from the collection plates using a wet paintbrush and transferred to fresh agar plates with a source of wet yeast paste with (Y + S) or without (Y) 20% sucrose. At 24-hour intervals for ten or more consecutive days, the number of living/dead animals was counted and living animals scored for larval stage (1^st^, 2^nd^, 3^rd^ instar). Living larvae were transferred to fresh plates every two days. Larval stages were determined by mouth hook or anterior and posterior spiracle morphology. 30 animals were used per plate and each treatment performed in triplicate. Standard deviation between the three replicates was calculated for the respective assays.

### Larval feeding assay

Twenty-four hours after the embryo collection, 1^st^ instar larvae were picked from embryo collection plates using a wet paintbrush and placed on fresh agar plates with yeast paste alone (Y) or yeast paste supplemented with 20% sucrose (Y + S). Forty-eight hours later the number of larvae outside the food source were counted under a dissection microscope (Leica S6E). All feeding experiments were done at room temperature (~23 °C) using 30 animals per plate and each treatment (Y or Y + S) performed in triplicate. Standard deviation between the three replicates was calculated.

### Larval denticle belt analysis

2^nd^ instar larvae were picked from agar plates with a wet paintbrush, placed on a CO_2_ block and examined under a dissection microscope (Leica S6E) for denticle belt defects. Those with severe defects were heated briefly at 60 °C (to kill and elongate the larvae) and imaged with a dissection microscope (Leica MZ10F equipped with a QImaging MicroPublisher 3.3 RTV camera and Q-Capture Pro 7 software).

### *dMegf8* knockdown via RNAi

*For in vivo* RNAi knockdown of *dMegf8*, the Gal4-UAS system^31^ was used. A transgenic fly line carrying a *UAS-RNAi* construct targeting the *Drosophila MEGF8* orthologue *CG7466* (*UAS-RNAi-dMegf8*) was obtained from the Vienna *Drosophila* Resource Centre (VDRC, stock #8018). As the inducible *UAS-RNAi* responder construct in this stock is inserted on the X (1^st^) chromosome of male flies only, we crossed *UAS-RNAi-dMegf8* males to virgin female flies from selected Gal4 driver lines (see below). Given that only female progeny inherit the X chromosome from the male parent, only female offspring from this cross were affected by the RNAi.

Virgin females from the following Gal4 driver lines were crossed to *UAS-RNAi-dMegf8* males: *Tubulin-Gal4/TM3, Sb* (BL #5138), *Actin5C-Gal4/CyO* (BL #4414), *MS1096-Gal4* (BL #8860), *elav*^*c155*^*-Gal4* (BL #458), *how*^24B^*-Gal4* (BL #1767), *Mef2-Gal4* (BL #27390), *Dll-Gal4*, *GMR-Gal4* (provided by I. Davis, University of Oxford, Oxford), *Sgs3-Gal4* (BL #6870), *btl-Gal4* (BL #8807). Male and female progeny that inherited a balancer chromosome from the Gal4 parents were excluded from analysis. In the control cross, wild-type virgin females were used in place of the Gal4 driver.

To test for a statistically significant difference between the means of the crosses, a one-way ANOVA was performed.

### Data availability

All data generated or analysed during this study are included in this published article (and its Supplementary Information files).

## Electronic supplementary material

Supplementary Information

Dataset 1
