# Supplementary material for: The Drosophila homologue of MEGF8 is essential for early development
Source: Sci Rep. 2018 Jun 8;8:8790. doi: 10.1038/s41598-018-27076-y (PMC5993795; doi:10.1038/s41598-018-27076-y)
Supplement: Supplementary file 1 — Supplementary Information [file 41598_2018_27076_MOESM1_ESM.docx]

**Supplementary Information**

**The *Drosophila* homologue of *MEGF8* is essential for early development**

**Deborah L. Lloyd, Markus Toegel, Tudor A. Fulga* and Andrew O.M. Wilkie***

**Supplementary Information 1: Oligonucleotides**

**Guides for CRISPR-Cas9-mediated gene editing**

| **Name** | **Forward (5'- 3')** | **Reverse (5'- 3')** |
| --- | --- | --- |
| CRISPR1-pAC | TTCGCAAATTTTTGGCTATCTAAC | AACGTTAGATAGCCAAAAATTTGC |
| CRISPR2-pAC | TTCGCACCAGCGTCCAGAATGTCA | AACTGACATTCTGGACGCTGGTGC |
| CRISPR3-pAC | TTCGGCGCCGACGGCGATGTGGA | AACTCCACATCGCCGTCGGCGCC |

**Colony PCR and sequence validation of guide insertion into plasmids**

| **Name** | **Forward (5'- 3')** | **Reverse (5'- 3')** | **Amplicon size (bp)** | **Annealing Tm (^o^C)** |
| --- | --- | --- | --- | --- |
| pAC-sgRNA backbone FW +  U6 PCR RV | GATCTAAAAAAGCACCGACTCGGTGCCACT | GACTTGCAGCCTGAAATACGGCACGAGT | 501 | 63 |
| CFD3 U6:3 Colony PCR FW +  CRISPR2-pCFD3 RV | CCAAGAGGCGAAAAGGTTAGC | AAACTGACATTCTGGACGCTGGTG | 144 | 56 |
| CFD3 U6:3 SEQ FW +  CFD3-sgRNA backbone RV | GCAAAAAAGCACCGACTCGGTGCCACT | CTACTCAGCCAAGAGGCGAAAAGGTTAGC | 236 | 63 |

**CRISPR-Cas9 mutation detection and/or sequence validation via HRMA**

| **Name** | **Forward (5'- 3')** | **Reverse (5'- 3')** | **Amplicon size (bp)** | **Annealing Tm (^o^C)** |
| --- | --- | --- | --- | --- |
| HRMA 1 | CCAGCGGCAAGTCGTGAGCAATG | GGGTGGGCCTGCTCTGTTTGCA | 193 | 60 |
| HRMA 2 | GGCCAAAACGGCAACCAGTTAGATAGC | ATTTCGCCGTAGGGCTCCGTGAAC | 198 | 60 |
| HRMA 3 | GATTAAAATGCAGAAAATGTACGCGTTGCTAG | CGTGTAGTTAAAGCCGGAGGGTCCAT | 178 | 60 |

**dMegf8 mutation validation in flies**

| **Name** | **Forward (5'- 3')** | **Reverse (5'- 3')** | **Amplicon size (bp)** | **Annealing Tm (^o^C)** |
| --- | --- | --- | --- | --- |
| CRISPR Mutant Check FW/RV | AGCGATAGGCGGCGACAAGCAACGT | ccactcgtcaaggcaaaacacaacttacACT | 671 | 62 |
